# Caecum Rupture Secondary to Iliac Crest Bone Graft: A Case Report

**DOI:** 10.7759/cureus.49545

**Published:** 2023-11-28

**Authors:** Wangsheng Wu, Bingsheng Liu, Huajuan Wang

**Affiliations:** 1 Orthopaedics, The Quzhou Affiliated Hospital of Wenzhou Medical University, Quzhou People's Hospital, Quzhou, CHN; 2 Orthopaedics, The Affiliated Tumor Hospital of Xinjiang Medical University, Urumqi, CHN; 3 Anaesthesiology, The Quzhou Affiliated Hospital of Wenzhou Medical University, Quzhou People's Hospital, Quzhou, CHN

**Keywords:** case report, caecum rupture, complications, autogenous bone harvesting, iliac crest bone graft

## Abstract

Autogenous bone grafting is a common surgical method in orthopaedics. The anterior iliac crest is a common site for harvesting autologous bone grafts. There are many complications after iliac bone harvesting, and pain and discomfort at the donor site are the most common sequelae. However, intestinal rupture after iliac bone harvesting has not been reported. We report a case of caecum rupture in a 58-year-old male after harvesting bone from his iliac crest. After proper surgical repair, the patient was discharged from the ICU and his bowel function recovered. This serious complication of bone harvesting from the iliac crest prompted investigation of the technique of iliac crest harvesting and donor site reconstruction.

## Introduction

The iliac crest is a common site for bone harvesting in orthopaedic surgery because a large amount of high-quality bone can be obtained there. The posterior iliac crest is often used as the donor site in spinal surgery, while the anterior iliac crest is often used as the donor site in limb surgery. Cancellous or cortical cancellous bone can easily be obtained from the anterior iliac crest, but given that it is a common donor site, the procedure has led to complications. Considerable morbidity regarding the iliac bone harvesting from the donor site has been widely reported in the literature, such as chronic pain, sensory loss, wound breakdown, contour defect, gait disturbance, pathologic fracture, adynamic ileus, ureteral injury, seroma, haematoma, haemorrhage, and hernia [1-6].

We report a case of intestinal rupture after iliac bone harvesting. To the best of our knowledge, this is the first case published in the literature.

## Case presentation

We report the case of a 58-year-old male, weighing 85 kg with a height of 175 cm suffering an open supracondylar femoral fracture. The patient underwent surgical treatment including open reduction and internal fixation. Due to large bone defect, a corticocancellous bone graft from the iliac crest was also placed. The graft was harvested from the bilateral iliac crest. Closure was performed using nonabsorbable sutures. There were no early postoperative complications, such as haematoma or infection at the donor site.

**Figure 1:**
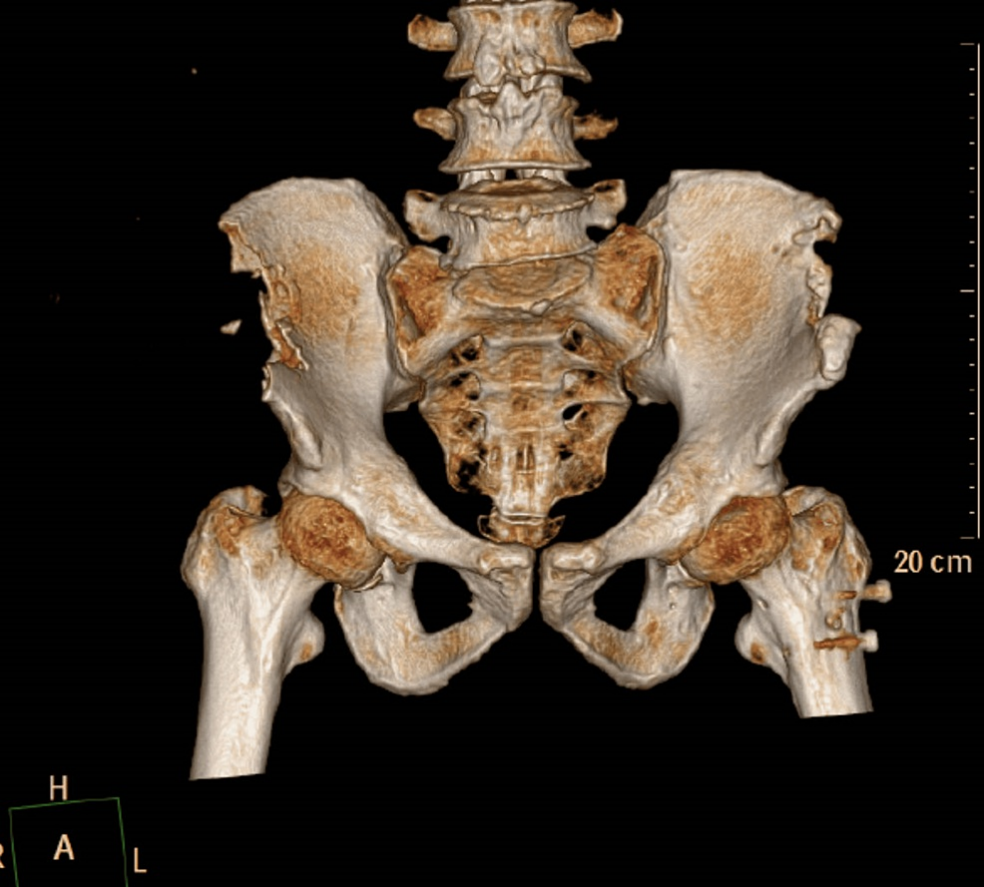
Three-dimensional CT reconstruction showing the postoperative state of bilateral iliac crest bone harvesting and the residual part of the right iliac crest was sharp

**Figure 2:**
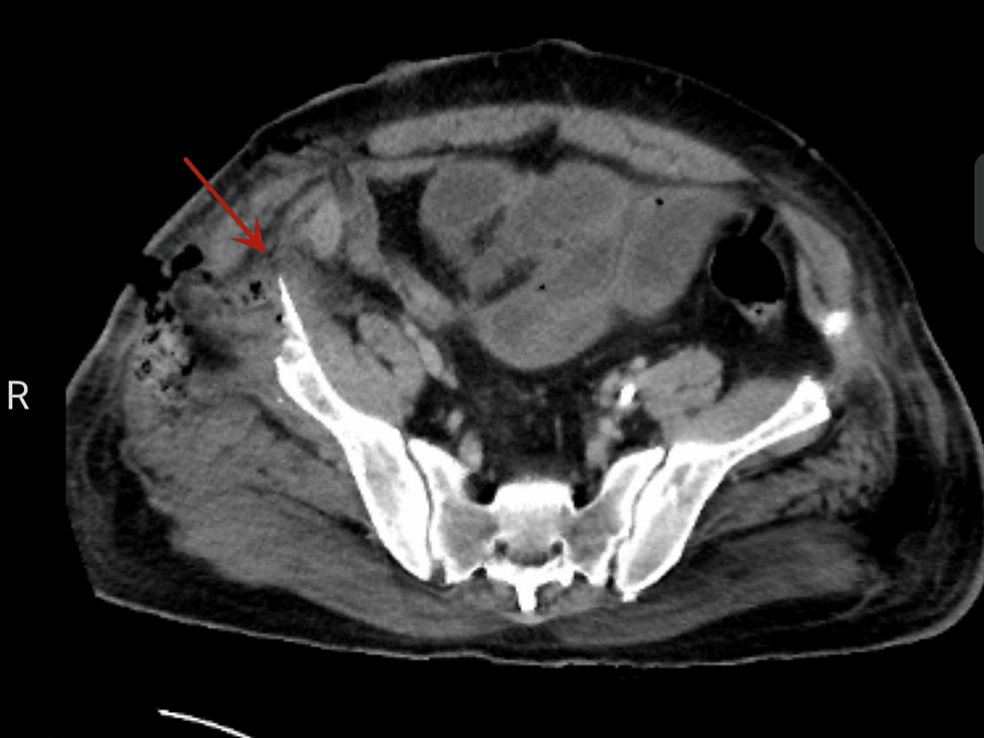
Abdominal CT scan (axial section) showing that there is gas in the right lower abdomen with the possibility of faecal accumulation

One month after this surgery the patient developed abdominal pain without obvious injury. Upon physical examination, there were obvious signs of peritoneal irritation: abdominal muscle tension, tenderness, and rebound tenderness. The patient complained about abdominal pain that was unbearable. The patient had no history of gastrointestinal disease or surgery. Therefore, the patient underwent an abdominal CT scan to investigate the abdominal pain. The imaging studies showed the postoperative state of bilateral iliac crest bone harvesting and the residual part of the right iliac crest was sharp and shaped like a dagger (Figure 1). There was pneumatosis in the right lower abdomen with the possibility of faecal accumulation, which suggests rupture of the bowel (Figure 2).

Laboratory tests showed that inflammatory markers were significantly increased: C-reactive protein (CRP) >200 mg/L. After a multidisciplinary discussion, emergency laparotomy was decided. Rupture of the peritoneum at the iliac fossa and an approximately 4 cm tear in the caecum were found during surgery. The orthopaedic surgeon entered along the original incision and found a large amount of pus and faecal water in the iliac fossa and the residual part of the iliac spine was sharp. The general surgeon repaired the peritoneum after thorough debridement, resected the ruptured caecum and performed enterostomy. After thorough debridement and extensive irrigation, the iliac crest was trimmed and covered with antibiotic-loaded cement. Two drainage tubes were placed in the abdomen and iliac fossa and then the incision was closed. The patient recovered satisfactorily postoperatively, and the abdominal and iliac fossa infections subsided.

## Discussion

To the best of our knowledge, we report the first case of caecum rupture as a complication of harvesting cortical cancellous bone from the anterior iliac crest. However, other abdominal complications have been reported, such as hernia and ureteral injury. Caecal rupture is an extremely serious complication that can lead to septic shock and even death. Moreover, the incidence of osteomyelitis increased significantly after the osteotomy site was soaked in faecal water. Fortunately, through the cooperation of our team and timely operation, the patient’s life was no longer threatened and the infection subsided.

Autogenous bone graft is the only clinically available bone graft material with osteogenesis, osteoinduction and osteoconduction functions and contains active precursor cells [7]. In addition, autologous iliac bone grafts have no immunogenicity, which ensures the stability of the grafts. There is also no risk of disease transmission with iliac crest autografts. The iliac crest is currently one of the most common sources of autologous bone. Since the introduction of autologous iliac bone grafting more than 60 years ago, the iliac crest has been recognized as the most suitable site for bone harvesting [7]. It has been clinically suggested that reliable bone union can be achieved by autogenous iliac bone grafting [7]. Therefore, an iliac bone graft in this case was the right choice. Iliac crest autografting is a difficult procedure, it causes complications. As early as 1989, it was shown in a study of 243 autogenous bone grafts in 239 patients who had complications including infection, massive haematoma, pain, sensory loss, and unsightly scars [8]. Summers et al. reported that significant pain at the donor site was reported in 33% of patients who received bicortical iliac bone grafts [9]. Eighty-eight per cent of patients who underwent tricortical full-thickness iliac bone harvesting complained of severe disabling pain that was stubborn and difficult to treat [9].

It is important to master the surgical techniques of iliac bone grafting and recognize postoperative complications to reduce complications at the donor site. The main reason for such a serious complication in the present case was that the stump was not trimmed after bone harvesting. The sharp bone end may cause unexpected damage to all surrounding soft tissues. A biomechanical study of the iliac crest after iliac bone harvesting showed that the bone harvesting site should be located at least 20 to 25 mm posterior to the anterior superior iliac spine to minimize the risk of bone fatigue in the iliac crest [10]. Pokharel et al. described an iliac crest harvesting technique that significantly reduced donor-site complications and achieved good postoperative results [11]. The study highlighted the following: (1) The skin incision was made after stretching the skin towards the abdomen to avoid the incision covering the bony prominent area, (2) The incision was started 3 cm posterior to the anterior superior iliac crest, (3) The iliac crest contour was preserved [11]. Ropars et al. conducted an autopsy combined with a radiology study on how to optimize iliac bone harvesting, and the study showed that donor site complications after iliac bone grafting could be reduced by improving the bone harvesting technique [12]. The recommended safe ranges for bone harvesting are as follows. The minimum safe distance from the anterior superior iliac spine (ASIS) to the anterior of the graft is 2 cm and could be increased to 3 cm. The graft should not exceed 35-mm depth and 45-mm length and should be situated anterior to the thickest part of the anterior iliac tubercle (AIT) [12]. Another point of consideration during harvesting is the use of a tool to make bony cuts. The use of an osteotome can cause microfracture of the donor site and graft, increase the risk of iliac fracture postoperatively, and reduce the strength of the graft. Jones et al. concluded that grafts taken with a pendulum saw were consistently stronger than grafts taken with a bone knife [13]. In addition, the use of a pendulum saw for bone harvesting allows precise control of the thickness/depth/direction of bone harvesting.

Many studies have shown that reconstruction of the bone defect in the donor site can reduce the morbidity of the donor site and achieve healing of the iliac bone defect and restoration of the bone contour [14-16]. In this case, after complete control of the infection, a second stage iliac bone repair was necessary because of the large size of the donor site defect to prevent further complications such as hernia. Gil-Albarova et al. suggested one-stage reconstruction of the bone defect after iliac bone harvesting [17]. A method of reconstruction is proposed in this paper: Additional small bone strips are taken from the donor site, and these extra small bone strips are designed to fill the bone defect area as a transverse fence. The clinical results showed that the reconstruction method was safe and effective [17]. Zhang et al. conducted a study of donor site repair after iliac bone grafting [18]. Studies have shown that reconstructing iliac crest defects after autogenous harvesting with bone cement and cancellous screws can reduce postoperative pain and improve cosmesis [18]. A study of surgical repair of hernias after tricortical iliac bone harvesting showed that reconstruction of the ilium with a titanium plate followed by peritoneal repair with partially absorbable mesh and fixation of the mesh to the titanium plate was effective in the treatment of hernias after tricortical iliac bone harvesting [19].

Considering these complications, we prefer to harvest bone grafts taken from the inner table and preserve the integrity of the outer plate. After the completion of bone harvesting, the stump of the donor site should be trimmed. Tricortical bone grafts are generally not taken. If it is necessary to harvest the tricortical bone due to the recipient site, then the stump should be trimmed after bone removal, and the donor site should be reconstructed in one stage.

## Conclusions

Caecum rupture after harvesting an iliac crest bone graft is a rare complication requiring emergency surgery. Fortunately, the patient's abdominal and donor site infections resolved. Therefore, before carrying out iliac crest bone graft harvesting, we should conduct repeated training and when deciding to perform iliac bone harvesting, we should be aware that any improper operation may cause postoperative complications.
